# Job Demands as Risk Factors of Exposure to Bullying at Work: The Moderating Role of Team-Level Conflict Management Climate

**DOI:** 10.3389/fpsyg.2019.02017

**Published:** 2019-09-04

**Authors:** Lena Zahlquist, Jørn Hetland, Anders Skogstad, Arnold B. Bakker, Ståle Valvatne Einarsen

**Affiliations:** ^1^Department of Psychosocial Science, Faculty of Psychology, University of Bergen, Bergen, Norway; ^2^Center of Excellence for Positive Organizational Psychology, Erasmus University Rotterdam, Rotterdam, Netherlands

**Keywords:** cognitive demands, conflict management climate, role conflict, workload, workplace bullying

## Abstract

Conflict management climate is an important organizational resource that is theorized to prevent interpersonal frustration from escalating into harsh interpersonal conflicts and even workplace bullying. The present study investigates whether team-level perceptions of conflict management climate moderate the relationship between previously investigated psychosocial predictors of workplace bullying (i.e., role conflicts, workload, cognitive demands) and perceived exposure to bullying behaviors in the workplace. We collected data from crews on ferries operating on the Norwegian coastline consisting of 462 employees across 147 teams. As hypothesized, multilevel analyses showed positive main effects of role conflict and cognitive demands (but not workload) on exposure to bullying behaviors. Also, the hypothesized moderation effect of team-level conflict management climate on the relationship between individual-level job demands and exposure to bullying behaviors was significant for role conflict and cognitive demands, but not for workload. Specifically, the positive relationships between the two job demands and exposure to bullying behaviors were stronger for employees working in teams with a weak (vs. a strong) conflict management climate. These findings contribute to the bullying research field by showing that conflict management climate may buffer the impact of stressors on bullying behaviors, most likely by preventing interpersonal frustration from escalating into bullying situations.

## Introduction

Although exposure to workplace bullying has been documented to be of a relatively low prevalence, it has shown to be a psychosocial stressor with severe negative consequences for the health and well-being of those targeted (Bowling and Beehr, 2006; Nielsen et al., 2014; Verkuil et al., 2015), as well as for the social environment where it occurs (Einarsen et al., 1994; Vartia, 2001). Despite extensive studies and knowledge about the detrimental outcomes of workplace bullying, including a long-term negative impact on mental health, increased risk for disability retirement, and personnel turnover, less is known about its possible risk factors (Baillien et al., 2009), and especially so regarding possible preventive factors that may influence the occurrence and the impact these risk factors may have on employee motivation, health, and well-being (Rai and Agarwal, 2018).

Among the risk factors that have been identified for workplace bullying, work-related strain factors are the most robust predictors (Bowling and Beehr, 2006). In accordance with the “work environment hypothesis” (Leymann, 1990, 1996; Einarsen et al., 1994), which claims that bullying is a consequence of work-related factors, previous studies have identified employees who have contradictory expectations and relatively high levels of job demands to be more often subjected to such bullying behaviors at work (Notelaers et al., 2010; Van den Brande et al., 2016; Nel and Coetzee, 2019). In line with this, job demands-resources (JD-R) theory states that every occupation and every job has specific demands and resources that in sum contribute to job-related stress or motivation (Bakker and Demerouti, 2017). A central assumption of JD-R theory is that, over time, high job demands may lead to strain and energy depletion (Bakker and Demerouti, 2007). Job strain, in turn, may lead to interpersonal frustration and bullying behaviors (Notelaers et al., 2013; Janssens et al., 2016). However, another central assumption of JD-R theory is that the presence of sufficient contextual and personal resources can buffer the energy depleting effects that high job demands potentially have (Bakker and Demerouti, 2007). Accordingly, such preventive resources may be job-related, such as autonomy, skill variety, and support from colleagues, or may be person-related, such as hardiness and self-efficacy. Resources may exist on different levels of the organization, and may also take the form of a conflict management climate in a specific department. A central assumption in the present study is therefore that conflict management climate constitute an important higher-level resource that may influence the potential job demands – bullying relationship.

Conflict management climate (CMC) refers to employees’ assessments of the organization’s conflict management procedures and practices, and of how fair and predictable the interactions between leaders and followers in this regard are perceived to be (Rivlin, 2001; Einarsen et al., 2018). In recent years, the concept of conflict management climate has gained growing interest as a promising mechanism, explaining why and when bullying occurs in a work environment. Bullying researchers have suggested and substantiated that conflict management climate is an important organizational resource that may prevent interpersonal frustration arising from stressful working conditions to escalate into workplace bullying (Einarsen et al., 2018). Since the concept of organizational climate has been defined as organizational members’ shared perceptions of a workplace phenomenon (James and James, 1989), we will apply a multilevel design with team-level perceptions of conflict management climate, also addressing the general request for more multilevel studies in the field of workplace bullying (Hauge et al., 2011; Skogstad et al., 2011). There is a strong need in the literature for adequate information from group level analyses in order to make appropriate interventions in groups and departments.

The aim of the present study is therefore to test the relationship between three identified individual level predictors of bullying (i.e., role conflict, workload, and cognitive demands), and reported exposure to bullying behaviors, yet add team-level conflict management climate in the equation. We will investigate whether this climate interacts with job stressors in predicting bullying-related outcomes. By integrating conflict management climate as a moderator, we aspire to obtain a more nuanced and better understanding of the antecedents and mechanisms explaining escalating bullying behaviors and the end-state of victimization from workplace bullying. In this, we address the general request for research on moderators in the job demands – bullying relationship (Rai and Agarwal, 2018), and also aspire to contribute valuable and nuanced knowledge on how to prevent workplace bullying from developing from other work-related stressors.

### Theoretical Background

Workplace bullying refers to the repeated and systematic exposure to negative behaviors in situations where the one targeted has difficulties defending him/herself in the actual situation (Einarsen et al., 2011). Hence, bullying is about the systematic mistreatment of a co-worker or a subordinate, often by psychological rather than physical means (Einarsen and Raknes, 1997; Keashly, 1997). The most frequently reported negative behaviors are withholding of information that affect the target’s work performance, having one’s opinions ignored, having key areas of responsibility removed or replaced with more trivial or unpleasant tasks, or being the target of spontaneous anger (Notelaers and Einarsen, 2013). Being a gradually escalating process, workplace bullying has shown to manifest itself in low as well as high intensities (Leon-Perez et al., 2012, 2015; Notelaers and Einarsen, 2013; Conway et al., 2018). Low-intensity bullying has been referred to as incivility or mistreatment at work (Cortina et al., 2001). In light of its preventive focus, the present study will investigate the whole range of exposure to bullying, from low intensity unwanted negative acts up to and including full-blown cases of victimization from bullying; conceptualized as exposure to bullying behaviors.

#### Situational Antecedents of Workplace Bullying

##### Role conflict

Role stressors, and particularly role conflict, represents one of the most studied and most important psychosocial risk factors at the workplace. Role conflict has consistently been found to predict reports of workplace bullying (Bowling and Beehr, 2006). Role conflict represents the simultaneous existence of two or more sets of expectations toward the same person, such that compliance with one set of expectations makes compliance with the other set difficult (Kahn et al., 1964; Beehr et al., 1995). Interestingly, role conflict was also one of the first work environment factors found to be linked to reports of exposure to workplace bullying (Einarsen et al., 1994; Vartia, 1996). Later studies have confirmed this relationship and identified role conflict to be among the strongest of all work-related predictors of workplace bullying (Hauge et al., 2007; Baillien and De Witte, 2009; Moreno-Jiménez et al., 2009). Accordingly, researchers have tried to theoretically explain why role conflicts are associated with workplace bullying. Einarsen et al. (1994) argue that the association between role conflict and workplace bullying is due to the creation of strain and frustration in the team, which may then elicit or fuel a bullying process. This aligns with JD-R theory, stating that role conflict is as a job demand that potentially can lead to energy-depleting strain (Bakker and Demerouti, 2007). Role conflict may also lead to frustration and stress in the focal person. Employees who experience role conflict become stressed, and may act in ways that irritate and annoy colleagues and superiors, and by that trigger a further process of incivility, interpersonal conflict, and mistreatment (Einarsen et al., 1994). This process is delineated in the extended “victim precipitation theory” (Elias, 1986; Samnani and Singh, 2016), proposing that when employees get stressed, they may act in ways that irritate and annoy colleagues and superiors, and by that trigger or fuel a bullying process. The following hypothesis is proposed:

*Hypothesis 1.* There is a positive relationship between role conflict and exposure to bullying behaviors at work.

##### Workload and cognitive demands

Next to role conflict, increased workload or work pressure has been suggested as an important precursor of bullying (Hauge et al., 2007; Baillien and De Witte, 2009). Although work pressure is a natural and necessary part of all working life, high work pressure over time, without sufficient recourses to cope with them, has been related to workplace bullying (Hauge et al., 2007; Parchment and Andrews, 2019). In fact, in the seminal work of Brodsky (1976), work pressure was proposed as a type of harassment by and in itself – when consistently being directed to one or more subordinates with the aim or likely outcome of punishing the target(s). However, the results of empirical studies have been mixed. While early studies failed to demonstrate such a relationship, more recent studies support the notion of a relationship between work pressure and bullying (Baillien and De Witte, 2009; Moreno-Jiménez et al., 2009). Quantitative demands, in the present study termed workload, have so far received most attention in research (Van den Brande et al., 2016). By workload, we refer to the amount and speed of work to be performed, which is whether you need to work fast or extra hard to get your tasks done (Van Veldhoven and Meijman, 1994). Niedl (1996) found, in his studies in Austria and Germany, a relationship between hectic work and reports of bullying at work. This finding has later been replicated in Norway (Hauge et al., 2007), Netherlands (Huber et al., 2001), and Belgium (Notelaers and De Witte, 2003).

Qualitative or cognitive demands, on the other hand, have received far less research attention. By cognitive demands, we refer to the need to concentrate one’s attention on several things at the same time, persistently be concentrated and careful in one’s work, or having many things to remember while conducting the work (Van Veldhoven and Meijman, 1994). Having high cognitive demands may be as stressful as time constraints and influences how one behaves and interacts with those around (Notelaers et al., 2010). Accordingly, Hoel et al. (2002) argue that cognitive demands are positively related to workplace bullying. They argue that workers under strain may voice their concern about the high cognitive demands, which may result in negative reactions and in some cases in conflict escalation, finally resulting in bullying (Baillien et al., 2009). Accordingly, Knorz and Zapf (1996) argued that high workload and cognitive demands can lead to conflict escalation, because those involved will have sparse time and limited resources for conflict resolution and management. As with role conflict, we expect in line with JD-R theory and victim precipitation theory, work pressure to be positively related to bullying behaviors. Thus, we propose:

*Hypothesis 2.* There is a positive relationship between workload and exposure to bullying behaviors at work.

*Hypothesis 3.* There is a positive relationship between cognitive demands and exposure to bullying behaviors at work.

#### Conflict Management Climate

Based on interviews with more than 1000 targets of work harassment, Brodsky (1976) claimed that for harassment to occur there needs to be a culture and climate that permits and rewards it. The concepts of organizational culture and climate offer to some extent overlapping perspectives for understanding the experiences people have in work settings (Denison, 1996), where organizational climate can be defined as organizational members’ shared perceptions of the workplace, in particular regarding its procedures, practices, prevailing behaviors, and its support and reward systems (James and James, 1989). In the present study, we will focus on the subjective perception of employees regarding how well the organization handles interpersonal conflicts based on their observations of how organizational procedures work in this area, of the habits managers have in such cases, as well as observations of consistent behaviors portrayed by managers when handling these kinds of interpersonal conflicts and claims of mistreatment. An element of trust is a natural ingredient in this and exchange of views and experiences between organization members will also to some extent shape the perceptions and attitudes involved. The perceptions are inherently subjective but are expected to be shared by those belonging to the same department or work group. To the extent that such perceptions are shared, we may talk about an organizational climate and not only a psychological climate, which again may affect the individual behavior and reactions of organization member, for example when involved in actual cases of interpersonal stress, frustration and escalating conflicts (James and Jones, 1980; Schneider et al., 1998). Such a climate may also be perceived as an organizational resource that affects the behaviors and reactions of employees and thus being consistent with the JD-R theory, proposing that the potential detrimental effect of job demands on the social relationships at work, may be prevented or litigated by resources in the organization and in the psychosocial working environment (Bakker and Demerouti, 2007). Although such job resources may be of a physical, psychological, social or organizational nature, organizational climate is proposed as a particularly strong resource in regard to interpersonal and social relations (Bakker and Demerouti, 2007).

In contemporary organizational research, it is common to study such specific types of climate, like climate for creativity, safety climate (Schneider, 2000), and in our case climate for conflict management. Hence, climate has an object, something we focus on, think off and act and react to. Regarding workplace bullying and prior empirical studies, some studies exist on the concept of psychosocial safety climate, with promising findings. In a recent longitudinal study, Dollard et al. (2017) found that a strong psychosocial safety climate predicted reduced bullying 4 years later, mediated by enacted psychosocial safety climate. These findings suggest that organizations with a strong psychosocial safety climate have a decreased likelihood of bullying through its influence on procedures implemented in the following three areas; (a) procedures directly addressing bullying; (b) procedures addressing reducing demands; and (c) procedures addressing the management of conflicts. In line with this perspective, Kwan et al. (2016) found that employees experiencing high psychosocial safety climate were more likely to choose an active coping strategy and voice bullying early, which prevented bullying incidents from further escalation.

Since conflict management climate is thought of as a sub facet of enacted psychosocial safety climate (Einarsen et al., 2018), we expect similar effects of conflict management climate on bullying. Consequently, we hypothesize that a strong conflict management climate, defined as employees’ beliefs that interpersonal conflicts are generally managed well and fairly in their organization (Rivlin, 2001), play an important role in preventing that a psychosocial work environment ripe with frustration poses a risk for workplace bullying. In a cross-sectional survey among employees in an on-shore transport company, Einarsen et al. (2018) found that conflict management climate was related to lower frequency reports of bullying as well as being a buffer in the bullying – work engagement relationship. The present study expands this research by testing whether perceived conflict management climate at the team-level can buffer the relationship between work-related factors and exposure to workplace bullying. We believe that the individual’s immediate work group is the primary group of interest in this regard, because this group in general is likely to exert more influence on the individuals involved than are larger more peripheral groups such as the entire organization (Bliese and Jex, 2002). On the background of JD-R theory and previous research, we propose that a strong conflict management climate, as a prevailing perception in the immediate work group, will buffer the impact of job demands on job strain, in our case perceived exposure to bullying behaviors. Hence, the three following hypotheses are presented:

*Hypothesis 4a.* The positive relationship between role conflict and bullying behaviors is moderated by conflict management climate. Specifically, the relationship between role conflict and exposure to bullying behaviors is weaker in teams with a strong (vs. weak) conflict management climate.

*Hypothesis 4b.* The positive relationship between workload and bullying behaviors is moderated by conflict management climate. Specifically, the relationship between workload and exposure to bullying behaviors is weaker in teams with a strong (vs. weak) conflict management climate.

*Hypothesis 4c.* The positive relationship between cognitive demands and bullying behaviors is moderated by conflict management climate. Specifically, the relationship between cognitive demands and exposure to bullying behaviors is weaker in team with a strong (vs. weak) conflict management climate.

## Materials and Methods

### Procedure and Sample

The present study was conducted using a sample of Norwegian employees in a major transport company, working on board ferries in regular service along the Norwegian coastline. As a part of a work environment survey, a questionnaire was distributed to 837 employees on all their ferries. Altogether, 462 questionnaires were returned, resulting in a response rate of 55.2%. The mean age of the sample was 45.04 years (SD = 11.77), ranging from 17 to 66 years, where 82% (*n* = 379) were males. The majority of the sample reported to be in a full time employment (93.2%). The sample was naturally clustered, as individual crew members belonged to teams sharing a particular captain, ferry and shift, creating a multi-level research design. The sample consisted of 147 teams with an average of 2.7 employees per team. Each vessel had 3–4 teams working in respective shifts, and each team consisted of a crew of 2–10 members.

The study was approved by the Norwegian Social Science Data Services/Norwegian Center for Research Data. An information letter was included with the request. Informing that participation was voluntary, that participants could resign from the study at any time, that the information provided would be threated confidentially and that the participants could ask later to have the information deleted.

### Instruments

*Exposure to bullying behaviors* at work was measured using the twelve-item version of the Negative Acts Questionnaire-Revised (“NAQ-R”; Einarsen et al., 2009; Glasø, 2010; Notelaers et al., 2018). The NAQ measures perceived exposure to bullying behaviors while at work, describing different kinds of behavior that may be perceived as bullying if they occur on a systematic and regular basis. The overall starting sentence was: “Which unwanted actions or negative situations have you been exposed to in your workplace during the last 6 months?” Example items are: “Someone withholding information which affects your performance,” “Spreading of gossip and rumors about you,” and “Being shouted at or being the target of spontaneous anger,” with response categories ranging from 1 (never) to 5 (daily). The scores on all items were summed to form an overall index of exposure to bullying behaviors. The scale showed good reliability, Cronbach’s α = 0.91.

*Role conflict* was measured using five items from the Role Questionnaire (Rizzo et al., 1970). An example item is: “I receive incompatible requests from two or more people,” with response categories ranging from 1 (very false) to 7 (very true). The scale showed adequate reliability, Cronbach’s α = 0.82.

*Workload* was measured using four items from the Questionnaire on the experience and assessment of work (Van Veldhoven and Meijman, 1994). An example item is: “Do you have to work very fast?” The response categories range from 1 (never) to 4 (always), and the scale showed good reliability, Cronbach’s α = 0.84.

*Cognitive demands* was measured using three items from the Questionnaire on the experience and assessment of work (Van Veldhoven and Meijman, 1994). An example item is: “Do you have to be attentive to many things at the same time?” The response categories range from 1 (never) to 4 (always), and the scale showed acceptable reliability, Cronbach’s α = 0.68.

*Conflict management climate* was measured with four items adapted from the Conflict Management Climate Scale regarding perceived fairness of dispute resolution in the organization (Rivlin, 2001; Einarsen et al., 2018). The wordings of the four items are as follows: (1) “If I have a serious disagreement with someone at work, I know who I should talk to about it”; (2) “The way we deal with disagreements between employees in my unit works well”; (3) “My superiors deal with conflicts in a good manner”; (4) “We have good procedures and methods for raising disagreements and conflicts in my workplace.” The response categories range from 1 (strongly disagree) to 5 (strongly agree). The scale showed good reliability, Cronbach’s α = 0.81. Prior to the multilevel analysis, the items were computed into a sum-score, and a team average score was used at the team-level in the analysis.

### Analyses

In order to acknowledge and analyze the multilevel structure of the data, implying that individual scores (individual-level) were nested within teams (team-level), we conducted multilevel analysis using MLwiN 2.20. In the analysis, individual-level predictors were centered on the team mean, while team-level predictors were centered on the grand mean. To test our hypotheses, we ran three models predicting bullying behaviors (NAQ-R). First, we tested a model where the intercept was included as the only predictor (Null Model). In the next model (Main effect Model), we included the explanatory demands variables (role conflict, workload, cognitive demands) and the moderator variable (conflict management climate). In the third model (Interaction Model), the two-way interactions between conflict management climate and the three demands were included. Simple slope tests for hierarchal linear models were used to examine whether the slopes in cross-level interactions were significantly different from zero (Preacher et al., 2006). The slopes where tested at ±1 SD for the predictors and moderators, and calculations were based on the asymptotic covariance matrix from the respective multilevel models using R version 3.4.3.

## Results

### Preliminary Confirmatory Factor Analyses

Prior to aggregating the conflict management climate scores to team-level, we performed a set of confirmatory factor analyses using Mplus 7.0 in order to assure that there is sufficient discriminant validity across the study constructs. In order to test this, we first modeled bullying behavior, role conflict, workload, cognitive demands and conflict management climate as five correlated latent factors using their respective observed indicators. The model showed acceptable fit (χ^2^ (df) = 887.24 (368), CFI = 0.91, TLI = 0.90, and RMSEA = 0.055), and revealed acceptable factor loadings in the range of 0.44 to 0.86. Moreover, correlations between the different latent constructs range from -0.47 to 0.50, all in the expected direction. Secondly, the constructs with the highest correlations (role conflict and bullying behaviors) where collapsed into one structure resolving in a four factor model. However, this resulted in a deteriorated fit (Δχ^2^ (Δdf) = 518.25 (4), *p* < 0.01, CFI = 0.81, TLI = 0.80, and RMSEA = 0.078). In sum, preliminary CFA analyses indicate that the constructs can be empirically distinguished.

### Descriptive Statistics

Means, standard deviations, Inter Class Correlations (ICC) for within-level variables, and within- and between-level correlations for all study variables are presented in Table 1. For conflict management climate, the estimated ICC2 (Bliese, 2000) was calculated to be 0.53. Correlational analysis showed that at the within-level, significant positive correlations between all three job demands and exposure to bullying behaviors, respectively, with the strongest relationship between role-conflict and exposure to bullying. Furthermore, role-conflict was positively related to workload, while workload was also positively related to cognitive demands. On the between-level, strong negative correlations exist between conflict management climate and bullying and role-conflict. Conflict management climate was not related to workload and cognitive demands.

**TABLE 1:** Mean, standard deviation, ICC, and within- and between-level correlations for all study variables (*N* = 462 participants, *N* = 147 teams).

	**$\bar{X}$**	**SD**	**ICC1/ICC2**	***S*^2^ between**	***S*^2^ within**	**1**	**2**	**3**	**4**
**Within-level**									
(1) Bullying behaviors	1.287	0.436	0.056^a^	0.021^∗^	0.135^∗∗^	–	0.402^∗∗^	0.136^∗^	0.131^∗^
(2) Role conflict	3.194	1.334	0.077^a^	0.170	1.578^∗∗^	0.887^∗∗^	–	0.209^∗∗∗^	0.102
(3) Workload	2.302	0.530	0.028^a^	0.007	0.248^∗∗^	0.729	0.655	–	0.353^∗∗^
(4) Cognitive demands	2.997	0.588	0.040^a^	0.015	0.327^∗∗^	0.137	–0.187	0.466	–
**Between-level**									
(5) CMC	3.749	0.611	0.535^b^	0.560^∗∗^	–	–0.957^∗∗^	–0.786^∗∗^	–0.658	–0.220

*CMC, conflict management climate; ^*a*^ICC1, within-level correlations; ^*b*^ICC2, between-level correlations; Correlations below the diagonal are correlations on the between-level. Correlations above the diagonal are correlations on the within-level. ^∗^*p* < 0.05, ^∗∗^*p* < 0.01, ^∗∗∗^*p* < 0.001.*

### Multilevel Analysis

As can be seen in Table 2, the initial unpredicted null model revealed that 3% of the total variance in bullying behaviors existed on the team-level while 97% of the variance appeared at the individual level. This suggests that most of the variance in bullying behaviors is explained by individual factors, rather than by team affiliation, which is consistent with our hypotheses trying to predict individual employees’ exposure to bullying behaviors. In hypotheses 1, 2, and 3, we hypothesized a positive association between (a) job demands in the form of role conflict, workload, and cognitive demands, and (b) exposure to bullying behaviors. In support of hypothesis 1 and 3, significant positive relationships were found for both role conflict (B = 0.103, *p* < 0.01) and cognitive demands (B = 0.105, *p* < 0.05) in the main effect model. Thus, when role conflicts or cognitive demands were higher, employees were more likely to report having been exposed to negative acts. However, the association between workload and bullying behaviors was not significant (B = 0.019, n.s.). Hence, hypothesis 2 was not supported. Finally, the main effect model reveals a significant negative relationship between conflict management climate and perceived bullying behaviors (B = -0.185, *p* < 0.05). This means that bullying behaviors are less likely in teams with a strong conflict management climate.

**TABLE 2:** Multilevel estimates for the prediction of bullying behaviors.

	**Null model**		**Main effect**		**Interaction**	
			**model**		**model**	
	***B***	**SE**	***B***	**SE**	***B***	**SE**
Intercept	1.272^∗∗^	0.020	1.275^∗∗^	0.018	1.275^∗∗^	0.018
Role conflict			0.103^∗∗^	0.018	0.101^∗∗^	0.017
Workload			0.019	0.047	–0.003	0.046
Cognitive demands			0.105^∗^	0.041	0.119^∗^	0.041
CMC			−0.185^∗^	0.028	−0.189^∗^	0.028
CMC × Role conflict					−0.071^∗^	0.033
CMC × Workload					0.060	0.078
CMC × Cognitive demands					−0.174^∗^	0.065
Variance level 1 (individual level)	0.145 (97%)	0.008	0.115	0.010	0.108	0.010
Variance level 2 (team-level)	0.004 (3%)	0.013	0.001	0.006	0.004	0.006
−2 Log likelihood	361.70		256.66		242.77	

*CMC, conflict management climate. *N* = 147 departments; *N* = 462 respondents. ^∗^*p* < 0.05, ^∗∗^*p* < 0.01.*

We further hypothesized, in hypotheses 4a, 4b, and 4c, that conflict management climate moderates the positive relationships between job demands and exposure to bullying behaviors. In support of hypotheses 4a and 4c, we found significant interactions between team-level conflict management climate and both role conflict (B = -0.071, *p* < 0.05) and cognitive demands (B = -0.174, *p* < 0.05) in the interaction model. However, the interaction effect between workload and conflict management climate was not significant (B = 0.060, n.s.). Hence, hypothesis 4b was not supported.

**FIGURE 1:**
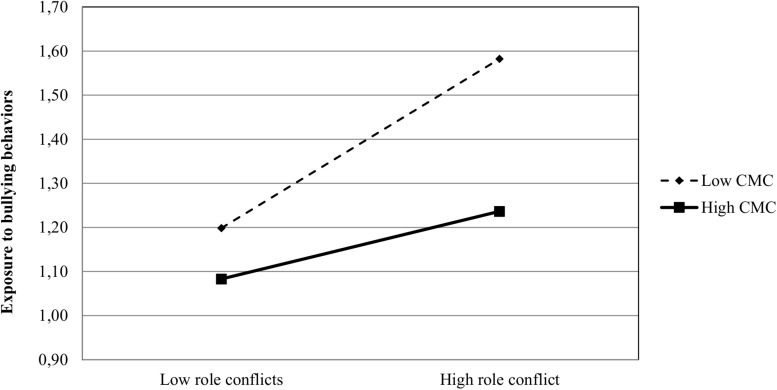
Plot of the interactive relationship of role conflict and bullying behaviors in teams with weak vs. strong CMC. CMC, conflict management climate.

**FIGURE 2:**
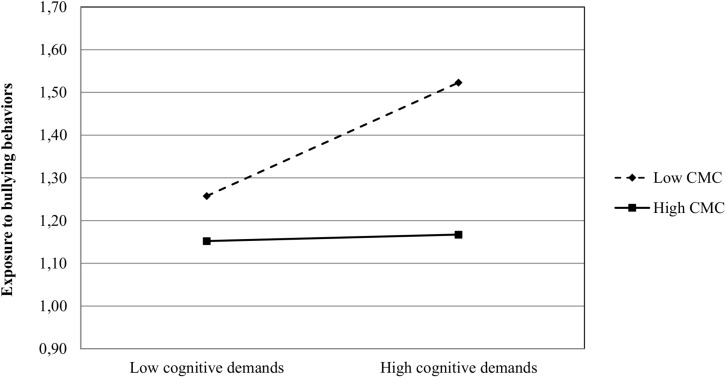
Plot of the interactive relationship of cognitive demands and bullying behaviors in teams with weak vs. strong CMC. CMC, conflict management climate.

The two significant interactions are visualized in Figures 1, 2. As can be seen in Figure 1, there is a stronger positive association between role conflict and exposure to bullying behaviors among respondents in teams characterized by a weak (i.e., low level) conflict management climate, compared to those working in teams with a strong (i.e., high level) conflict management climate. Despite these differences, a formal test of the slopes at ±1 SD of the moderator revealed significant slopes for both teams characterized by a weak conflict management climate (Slope = 0.058, z = 2.185, *p* < 0.05), and teams characterized by a strong conflict management climate (Slope = 0.144, z = 5.395, *p* < 0.01).

Inspection of Figure 2 reveals that while a clear positive effect between cognitive demands and exposure to bullying behaviors is found among individuals in teams with a weak conflict management climate, the slope among individuals in teams characterized by a strong conflict management climate is almost flat. Accordingly the simple slope test reveals a significant positive slope among those in teams with a weak conflict management climate (slope = 0.225, z = 3.631, *p* < 0.01), while the slope among individuals in teams with a strong conflict management climate (slope = 0.013, z = 0.248, n.s.) was not significant.

In order to rule out the possibility that the relationships can be explained by relevant third variables, we ran all the analyses while controlling for gender, age, and tenure. However, the analyses showed that none of the control variables significantly predicted exposure to bullying behavior. Based on this, we decided to only report the most parsimonious analyses excluding the control variables, in line with the suggestions of Cohen et al. (2013).

## Discussion

Based on the work environment hypothesis and job demands-resources theory, we hypothesized that role conflict, workload, and cognitive demands would be positively related to exposure to bullying behaviors at work. Psychosocial demands at work, such as role conflict, workload and cognitive demands are consistently found to predict experiences of being exposed to bullying behaviors in the workplace. In this study, we further examined to what extent team-level perceptions of conflict management climate buffer the potential relationship between these job demands and exposure to bullying behaviors at work. Being an organizational resource, conflict management climate provides workers with information on and confidence regarding where to go and what to do when strain arises, and frustration and conflicts appear. Furthermore, it provides guidelines of how to handle such situations and trust in the organization’s ability to act constructively if the situation would escalate. Hence, we predicted that the relationships between these stressors and exposure to bullying behaviors would be weaker in teams with a strong conflict management climate.

As hypothesized, the results of multilevel analyses showed positive main effects of role conflict and cognitive demands on exposure to bullying behaviors. Hence, employees who experience elevated levels of role conflict and cognitive demands in their work tend to report more exposure to bullying behaviors. In line with our findings, several studies have found that employees who experience high levels of role conflict and cognitive demands are more often exposed to bullying behaviors (Van den Brande et al., 2016). Our finding of role conflict as the most important predictor of workplace bullying aligns with previous research findings (Hauge et al., 2007; Notelaers et al., 2010). Across different professions, studies consistently find that role conflicts are a strong stressor (Einarsen et al., 1994; Hauge et al., 2007). In a Danish study, Agervold (2009) found that departments with the highest incidents of workplace bullying experienced more role conflicts and cognitive demands as compared to departments with the lowest incidents of bullying. The findings in the present study are therefore in support of the work environment hypothesis, which states that bullying is the result of stressors in the psychosocial working environment creating a fertile soil for frustration, irritation and accompanying episodes of interpersonal conflict (Leymann, 1990; Einarsen et al., 1994). These findings are also consistent with the extended victim precipitation theory, stating that when people get stressed, they are more likely to act in ways that provoke others, and by that evoke bullying behavior from potential perpetrators. Along similar lines, Salin (2003) argues that stress increases job dissatisfaction, lowers aggression thresholds and does not allow time for conflict solving. Additionally, the tendency to not take time for polite and friendly interactions at work when we are under stress (Pearson et al., 2000), can together with the other factors potentially increasing the risk for harsh and spiraling interpersonal conflicts, which may turn into bullying.

Contrary to our predictions and to previous research there was, however, no significant main effect of workload on employees’ exposure to bullying behaviors. In this regard, it is important to state that job demands are not necessarily something negative. LePine et al. (2005) distinguish between job demands as hindrance stressors and challenge stressors. They describe hindrance stressors as “bad” stressors that interfere with or inhibit an individual’s ability to achieve valued goals (Cavanaugh et al., 2000). While challenge stressors are considered as “good” stressors potentially promoting the personal growth and achievement of the employee. In line with this, workload has been termed as challenge stressors (Podsakoff et al., 2007). Although Van den Broeck et al. (2010) found support for the differentiation between challenge and hindrance demands, there is still not sufficient empirical evidence on this issue (Demerouti and Bakker, 2011). What one finds exhausting or not may also be dependent upon the said job.

The present study is, to our knowledge, the first to empirically demonstrate the buffering effect of conflict management climate on the link between these job demands and exposure to bullying behaviors. The results showed that a strong conflict management climate was related to lower reports of bullying behaviors in its own right as seen in the direct effect of climate and more so in the presence of role conflicts and cognitive demands. More specifically, the positive relationships between these job demands and bullying behaviors were stronger for employees working in teams with a weak conflict management climate. In line with recent research, our findings support that conflict management climate is an important organizational-level resource with the ability to prevent bulling both directly and indirectly by reducing the impact of other known risk factors (Einarsen et al., 2018).

Initially, it seems apparent that a strong conflict management climate contributes to the actual handling of interpersonal frustration and conflicts at an early stage. Choosing an active coping strategy and voicing the conflict early has been found to prevent bullying from future escalation (Kwan et al., 2016). Kwan et al. (2016) found that workers who chose a more passive strategy and neglected the bullying were more likely to experience that the bullying escalated. Those who then chose to voice later in the process often still experienced unsuccessful outcomes. One reason for this could be that the bullying then had escalated too far. In addition, they found that the likelihood of choosing an active coping strategy was dependent on the climate, in their case psychosocial safety climate (Kwan et al., 2016). When psychosocial safety climate was high, workers felt safe to voice their concerns and by that initiate support from organization and management in order to resolve the bullying. It seems that active coping strategies, such as voice, are not likely to be effective unless the climate is right (Kwan et al., 2016). Considering the similarities between psychosocial safety climate and conflict management climate, we might expect that by establishing a strong conflict management climate, teams and organizations can potentially foster active coping strategies in the face of conflicts and by this reinforce a positive cycle.

Further, and as argued by Einarsen et al. (2018) it is conceivable that conflict management climate works by reducing insecurity and by promoting predictability and perceived control. Rivlin (2001) argue that a strong conflict management climate implies that employees perceive managers to intervene in conflicts that arise and the conflict management procedures of their organization to be fair. A strong conflict management climate also provides workers with confidence regarding where to go and what to do when conflicts appear. Increased perception of control can further increase the likelihood that demands, as conflicts, are handled and more easily coped with (Karasek, 1979). To perceive control in conflict situations can then reduce the likelihood of frustration evolving and becoming interpersonal conflicts. A strong conflict management climate may imply the trust that negative behavior will be addressed, thereby preventing and stopping such behavior which otherwise may happen under stress. The experience of social support might also be an explanatory mechanism, as impartial and respectful attitudes of superiors is an important aspect of the experience of organizational justice, which may further promote employees’ perception of social support in the workplace (Fujishiro and Heaney, 2009).

Although not explicitly hypothesized, we found that team-level conflict management climate, in addition to having a buffering effect, also had a main effect on bullying behaviors. This finding also contributes to validate the concept of conflict management climate, indicating that work environments characterized by a strong conflict management climate are characterized by fewer bullying behaviors and a lower risk of bullying, irrespectively of such stressors. Alternatively, the direct negative relationship between conflict management climate and bullying may mean that environments with few bullying behaviors contribute to the perception of a strong conflict management climate.

### Practical Implications

The results of the present study have important practical implications for HR personnel, managers, and leaders, as the findings from this study indicate that developing teams and organizations characterized by strong conflict management climate can be beneficial in order to prevent destructive conflicts and bullying. This knowledge should be taken into consideration when developing evidence-based prevention-focused interventions. Taking into consideration the potential costs of bullying being related to reduced productiveness, and increased likelihood of sickness absence and turnover (Sheehan et al., 2001), preventive interventions are considered to be far more cost-effective than strategies that aim to repair the consequences of bullying (Rivlin, 2001). Further, interventions should be directed against factors in the organization, like job demands or climate, as factors in the work environment have consistently been found to be strong antecedents of workplace bullying (Einarsen et al., 1994; Van den Brande et al., 2016). The finding that such a climate moderates more than one risk factor indicates that focusing on conflict management climate may be particularly efficient as a preventive measure. However, we still find a relationship between role conflict and bullying behavior in teams with strong conflict management climate. This supports the notion that role conflicts are the strongest psychosocial predictor of workplace bullying, and stresses the need to simultaneously continue to enhance role clarity.

Furthermore, one advantage of studying specific climate dimensions is that actions targeted at addressing these elements of organizational climate are more manageable and effective, than actions more broadly focused (Giorgi, 2009). It is the management’s responsibility to create such a climate that is responsive to these interpersonal issues, hence, the focal group to address here is leaders. As climate can actively be shaped by people with power and influence (James and James, 1989), leaders should be trained in conflict management procedures. They should then communicate to their employees directions for whom they should contact and which actions to take if they are involved in disputes and conflicts, as well as how management will act to solve such cases (see also Einarsen and Hoel, 2008). Establishing clear guidelines for what to do when conflict occur can foster security and self-control. Implementation of such procedures may further promote the experience of fair conflict management when disputes and conflicts develop. These interventions should then be directed groups and departments in the organization, as our results show that conflict management do exist on team-level.

Taken together, the findings of the present study provide additional support to the well-established link between psychosocial factors, such as role conflict and cognitive demands, and the risk for exposure to bullying behaviors. Yet, and more interestingly, our findings demonstrate that the effect of these risk-factors may be alleviated or even eliminated by organizational teams’ or departments’ ability to manage conflicts and employees’ trust in this. As such, our findings have important theoretical and practical implications.

### Strengths and Limitations

A strength of the present study is the use of recognized scales with satisfactory validity and reliability. The accidental finding that conflict management climate is directly related to less reports of exposure to bullying speaks to the validity of the scale. Further, a considerable strength of the present study is that we measured conflict management climate at the appropriate level, as the concept of organizational climate is defined as organizational members’ shared perceptions of the workplace and therefore ideally exist on a group level (James and James, 1989). Integrating multilevel constructs can help capture the complexity of organizational phenomena and develop more sophisticated theoretical models (Demerouti and Bakker, 2011). There is, however, a further need for validating our findings in other work contexts. Regarding future research, it would be interesting to investigate the role of conflict management climate in other antecedents – workplace bullying relationships, as well as looking more closely at the involved mechanisms.

However, some limitations of the present study need to be considered. First, the study is based on cross sectional data, which means that all the information was collected at the same time. Causal relationships can therefore not be drawn based on our findings. Longitudinal studies are necessary in order to confirm the direction of the relationships between the studied variables. Another possible limitation of the current study is the problem of common method variance. Because we only use self-report questionnaires, we cannot rule out that some associations are biased by common method. Nevertheless, we do not expect this to be a prominent problem in our study as common method bias generally decreases when studying interactions (Siemsen et al., 2010).

Further, we encourage some caution when generalizing our results. The sample in our study consist of crews on ferries in a Norwegian transport company. Thus, the findings are not necessarily generalizable to all other occupational groups, as there may be factors in this work context that is not typical for all workplaces, influencing the results. For instance, the fact that these teams live closely together for 2–7 days in a row, could conceivably create a greater need for a strong conflict management climate. On the other hand, they also have longer periods off work, which could potentially make it harder to establish such a team-climate.

Lastly, it should be mentioned that the sample we chose had some clear advantages in regard to studying climate at a group level. These teams work together in fixed shifts, often for several days in a row, living, working, and sleeping at the ferry, which offers a unique opportunity for control when measuring teams. In most companies, it would be more difficult to measure the actual climate in the team, as it is common that employees work across teams, or even belong to several teams, making it hard to measure the climate variable.

## Conclusion

The present study was conducted for both theoretical, methodological and applied reasons and with findings with important implications. First, it provides a new and broader theoretical understanding of organizational risk factors and typical antecedents of workplace bullying in its focus on how conflict management climate buffer the relationships between job demands and workplace bullying. Methodologically, it is important as it answer a call in the literature for multilevel designs in the study of workplace bullying and further substantiate the usefulness of such a design. In terms of practice, we proposed a new factor within the work environment hypothesis which can be addressed by practitioners, and which may have both direct and indirect preventive effects. In this, our findings show that conflict management climate may serve as an important preventive tool against workplace bullying.

## Data Availability

The datasets generated for this study are available on request to the corresponding author. Any inquiries regarding the dataset can be addressed to Ståle Einarsen (stale.einarsen@uib.no).

## Ethics Statement

This study was approved by the Norwegian Social Science Data Services/Norwegian Centre for Research Data. An information letter was included with the request, informing that participation was voluntary, that participants could resign from the study any time and that the participants could ask later to have the information deleted. Thus, the response itself was seen as an informed consent.

## Author Contributions

All authors have been responsible for the study concept and design, actively involved in the writing process, and are collectively responsible for the final completion of the manuscript.

## Conflict of Interest Statement

The authors declare that the research was conducted in the absence of any commercial or financial relationships that could be construed as a potential conflict of interest.

## References

[B1] Agervold M. (2009). The significance of organizational factors for the incidence of bullying. *Scand. J. Psychol.* 50 267–276. 10.1111/j.1467-9450.2009.00710.x 19298225

[B2] Baillien E. De Witte H. (2009). Why is organizational change related to workplace bullying? Role conflict and job insecurity as mediators. *Econ. Ind. Democracy* 30 348–371. 10.1177/0143831x09336557

[B3] Baillien E. Neyens I. De Witte H. De Cuyper N. (2009). A qualitative study on the development of workplace bullying: towards a three way model. *J. Community Appl. Soc. Psychol.* 19 1–16. 10.1002/casp.977

[B4] Bakker A. B. Demerouti E. (2007). The job demands-resources model: state of the art. *J. Manag. Psychol.* 22 309–328. 10.1108/02683940710733115

[B5] Bakker A. B. Demerouti E. (2017). Job demands–resources theory: taking stock and looking forward. *J. Occup. Health Psychol.* 22 273–285. 10.1037/ocp0000056 27732008

[B6] Beehr T. A. Johnson L. B. Nieva R. (1995). Occupational stress: coping of police and their spouses. *J. Organ. Behav.* 16 3–25. 10.1002/job.4030160104 8559888

[B7] Bliese P. D. (2000). “Within-group agreement, non-independence, and reliability: implications for data aggregation and analysis,” in *Multilevel Theory, Research, and Methods in Organizations: Foundations, Extensions, and New Directions*, eds Klein K. J. Kozlowski S. W. J. (San Francisco, CA: Jossey-Bass).

[B8] Bliese P. D. Jex S. M. (2002). Incorporating a mulitilevel perspective into occupational stress research: theoretical, methodological, and practical implications. *J. Occup. Health Psychol.* 7 265–276. 10.1037//1076-8998.7.3.265 12148957

[B9] Bowling N. A. Beehr T. A. (2006). Workplace harassment from the victim’s perspective: a theoretical model and meta-analysis. *J. Appl. Psychol.* 91 998–1012. 10.1037/0021-9010.91.5.998 16953764

[B10] Brodsky C. M. (1976). *The Harassed Worker.* Toronto: Lexington Books: DC Heath & Co.

[B11] Cavanaugh M. A. Boswell W. R. Roehling M. V. Boudreau J. W. (2000). An empirical examination of self-reported work stress among US managers. *J. Appl. Psychol.* 85 65–74. 10.1037//0021-9010.85.1.65 10740957

[B12] Cohen J. Cohen P. West S. G. Aiken L. S. (2013). *Applied Multiple Regression/Correlation Analysis for the Behavioral Sciences.* Abingdon: Routledge.

[B13] Conway P. M. Høgh A. Nabe-Nielsen K. Grynderup M. B. Mikkelsen E. G. Persson R. (2018). Optimal cut-off points for the short-negative act questionnaire and their association with depressive symptoms and diagnosis of depression. *Ann. Work Expo. Health* 62 281–294. 10.1093/annweh/wxx105 29304192

[B14] Cortina L. M. Magley V. J. Williams J. H. Langhout R. D. (2001). Incivility in the workplace: incidence and impact. *J. Occup. Health Psychol.* 6 64–80. 10.1037//1076-8998.6.1.64 11199258

[B15] Demerouti E. Bakker A. B. (2011). The job demands-resources model: challenges for future research. *SA J. Ind. Psychol.* 37 01–09. 10.4102/sajip.v37i2.974

[B16] Denison D. R. (1996). What is the difference between organizational culture and organizational climate? A native’s point of view on a decade of paradigm wars. *Acad. Manag. Rev.* 21 619–654. 10.2307/258997

[B17] Dollard M. F. Dormann C. Tuckey M. R. Escartín J. (2017). Psychosocial safety climate (PSC) and enacted PSC for workplace bullying and psychological health problem reduction. *Eur. J. Work Organ. Psychol.* 26 844–857. 10.1080/1359432x.2017.1380626

[B18] Einarsen S. Hoel H. (2008). “Bullying and mistreatment at work: how managers may prevent and manage such problems,” in *Employee Well-Being Support: A Workplace Resource*, eds Kinder A. Hughes R. Cooper C. L. (Hoboken, NJ: John Wiley & Sons).

[B19] Einarsen S. Hoel H. Notelaers G. (2009). Measuring exposure to bullying and harassment at work: validity, factor structure and psychometric properties of the negative acts questionnaire-revised. *Work Stress* 23 24–44. 10.1080/02678370902815673

[B20] Einarsen S. Hoel H. Zapf D. Cooper C. L. (2011). “The concept of bullying and harassment at work: the european tradition,” in *Bullying and Harassment in the Workplace: Developments in Theory, Research, and Practice*, 2nd Edn eds Einarsen S. Hoel H. Zapf D. Cooper C. L. (Boca Raton: CRC Press).

[B21] Einarsen S. Raknes B. I. (1997). Harassment in the workplace and the victimization of men. *Violence Vict.* 12 247–263. 10.1891/0886-6708.12.3.247 9477540

[B22] Einarsen S. Raknes B. I. Matthiesen S. B. (1994). Bullying and harassment at work and their relationships to work environment quality: an exploratory study. *Eur. J. Work Organ. Psychol.* 4 381–401. 10.1080/13594329408410497

[B23] Einarsen S. Skogstad A. Rørvik E. Lande A. B. Nielsen M. B. (2018). Climate for conflict management, exposure to workplace bullying and work engagement: a moderated mediation analysis. *Int. J. Hum. Res. Manag.* 29 549–570. 10.1080/09585192.2016.1164216

[B24] Elias R. (1986). *The Politics of Victimization: Victims, Victimology, and Human Rights.* New York: Oxford University Press.

[B25] Fujishiro K. Heaney C. A. (2009). Justice at work, job stress, and employee health. *Health Educ. Behav.* 36 487–504. 10.1177/1090198107306435 18006665

[B26] Giorgi G. (2009). Workplace bullying risk assessment in 12 Italian organizations. *Int. J. Workplace Health Manag.* 2 34–47. 10.1108/17538350910945992

[B27] Glasø L. Vie T. L. Holmdal G. R. Einarsen S. (2010). An application of affective events theory to workplace bullying. *Eur. Psychol.* 16 198–208. 10.1027/1016-9040/a000026

[B28] Hauge L. J. Einarsen S. Knardahl S. Lau B. Notelaers G. Skogstad A. (2011). Leadership and role stressors as departmental level predictors of workplace bullying. *In. J. Stress Manag.* 18 305–323. 10.1037/a0025396

[B29] Hauge L. J. Skogstad A. Einarsen S. (2007). Relationships between stressful work environments and bullying: results of a large representative study. *Work Stress* 21 220–242. 10.1080/02678370701705810

[B30] Hoel H. Zapf D. Cooper C. L. (2002). “Workplace bullying and stress,” in *Historical and Current Perspectives on Stress and Health*, 2nd Edn eds Perrewe P. L. Ganster D. C. (Bingley: Emerald Group Publishing Limited).

[B31] Huber A. Furda J. Steensma H. (2001). Mobbing: systematisch pestgedrag in organisaties (mobbing: systematic harassment in organisations). *Gedrage Organisatie* 14 378–396.

[B32] James L. A. James L. R. (1989). Integrating work environment perceptions: explorations into the measurement of meaning. *J. Appl. Psychol.* 74 739–751. 10.1037//0021-9010.74.5.739

[B33] James L. R. Jones A. P. (1980). Perceived job characteristics and job satisfaction: an examination of reciprocal causation. *Pers. Psychol.* 33 97–135. 10.1111/j.1744-6570.1980.tb02167.x

[B34] Janssens H. Braeckman L. De Clercq B. Casini A. De Bacquer D. Kittel F. (2016). The indirect association of job strain with long-term sickness absence through bullying: a mediation analysis using structural equation modeling. *BMC Public Health* 16:851. 10.1186/s12889-016-3522-y 27549206 PMC4994183

[B35] Kahn R. L. Wolfe D. M. Quinn R. P. Snoek J. D. Rosenthal R. A. (1964). *Organizational Stress: Studies in Role Conflict and Ambiguity.* Oxford: John Wiley.

[B36] Karasek R. A. (1979). Job demands, job decision latitude, and mental strain: implications for job redesign. *Adm. Sci. Q.* 24 285–308. 10.2307/2392498 30315367

[B37] Keashly L. (1997). Emotional abuse in the workplace: conceptual and empirical issues. *J. Emot. Abuse* 1 85–117. 10.1300/j135v01n01_05

[B38] Knorz C. Zapf D. (1996). Mobbing - eine extreme form sozialer stressoren am arbeitsplatz. *Zeitschrift für Arbeits Organ. Psychol.* 40 12–21.

[B39] Kwan S. S. M. Tuckey M. R. Dollard M. F. (2016). The role of the psychosocial safety climate in coping with workplace bullying: a grounded theory and sequential tree analysis. *Eur. J. Work Organ. Psychol.* 25 133–148. 10.1080/1359432x.2014.982102

[B40] Leon-Perez J. Arenas A. Butts T. (2012). “Effectiveness of conflict management training to prevent workplace bullying,” in *Workplace bullying: Symptoms and Solutions*, ed. Tehrani N. (London: Routledge).

[B41] Leon-Perez J. M. Medina F. J. Arenas A. Munduate L. (2015). The relationship between interpersonal conflict and workplace bullying. *J. Manag. Psychol.* 30 250–263. 10.1108/JMP-01-2013-0034

[B42] LePine J. A. Podsakoff N. P. LePine M. A. (2005). A meta-analytic test of the challenge stressor–hindrance stressor framework: an explanation for inconsistent relationships among stressors and performance. *Acad. Manag. J.* 48 764–775. 10.5465/amj.2005.18803921

[B43] Leymann H. (1990). Mobbing and psychological terror at workplaces. *Violence Vict.* 5 119–126. 10.1891/0886-6708.5.2.119 2278952

[B44] Leymann H. (1996). The content and development of mobbing at work. *Eur. J. Work Organ. Psychol.* 5 165–184. 10.1080/13594329608414853 12350256

[B45] Moreno-Jiménez B. Rodríguez-Muñoz A. Pastor J. C. Sanz-Vergel A. I. Garrosa E. (2009). The moderating effects of psychological detachment and thoughts of revenge in workplace bullying. *Pers. Ind. Dif.* 46 359–364. 10.1016/j.paid.2008.10.031

[B46] Nel E. Coetzee M. (2019). Job demands–resources and flourishing: exploring workplace bullying as a potential mediator. *Psychol. Rep.* [Epub ahead of print]. 31027472 10.1177/0033294119839032

[B47] Niedl K. (1996). Mobbing and well-being: economic and personnel development implications. *Eur. J. Work Organ. Psychol.* 5 239–249. 10.1080/13594329608414857

[B48] Nielsen M. B. Magerøy N. Gjerstad J. Einarsen S. (2014). Workplace bullying and subsequent health problems. *Tidsskr. Nor. Laegeforen.* 134 1233–1238. 10.4045/tidsskr.13.0880 24989201

[B49] Notelaers G. Baillien E. De Witte H. Einarsen S. Vermunt J. K. (2013). Testing the strain hypothesis of the demand control model to explain severe bullying at work. *Econ. Ind. Democracy* 34 69–87. 10.1177/0143831X12438742

[B50] Notelaers G. De Witte H. (2003). Over de relatie tussen pesten op het werk en werkstress. *Paper Presented at the De arbeidsmarkt in Verslagboek Arbeidsmarktonderzoekersdag*, Vlaanderen.

[B51] Notelaers G. De Witte H. Einarsen S. (2010). A job characteristics approach to explain workplace bullying. *Eur. J. Work Organ. Psychol.* 19 487–504. 10.1080/13594320903007620

[B52] Notelaers G. Einarsen S. (2013). The world turns at 33 and 45: defining simple cutoff scores for the negative acts questionnaire–revised in a representative sample. *Eur. J. Work Organ. Psychol.* 22 670–682. 10.1080/1359432x.2012.690558

[B53] Notelaers G. Van der Heijden B. Hoel H. Einarsen S. (2018). Measuring bullying at work with the short-negative acts questionnaire: identification of targets and criterion validity. *Work Stress* 33 58–75. 10.1080/02678373.2018.1457736

[B54] Parchment J. Andrews D. (2019). The incidence of workplace bullying and related environmental factors among nurse managers. *JONA J. Nurs. Adm.* 49 132–137. 10.1097/NNA.0000000000000726 30789556

[B55] Pearson C. M. Andersson L. M. Porath C. L. (2000). Assessing and attacking workplace incivility. *Organ. Dyn.* 29 123–137. 10.1016/s0090-2616(00)00019-x

[B56] Podsakoff N. P. LePine J. A. LePine M. A. (2007). Differential challenge stressor-hindrance stressor relationships with job attitudes, turnover intentions, turnover, and withdrawal behavior: a meta-analysis. *J. Appl. Psychol.* 92 438–454. 10.1037/0021-9010.92.2.438 17371090

[B57] Preacher K. J. Curran P. J. Bauer D. J. (2006). Computational tools for probing interactions in multiple linear regression, multilevel modeling, and latent curve analysis. *J. Educ. Behav. Stat.* 31 437–448. 10.3102/10769986031004437

[B58] Rai A. Agarwal U. A. (2018). A review of literature on mediators and moderators of workplace bullying: agenda for future research. *Manag. Res. Rev.* 41 822–859. 10.1108/mrr-05-2016-0111

[B59] Rivlin J. N. (2001). *Conflict Management Climate Related to Employment Litigation.* Atlanta: Georgia Institute of Technology.

[B60] Rizzo J. R. House R. J. Lirtzman S. I. (1970). Role conflict and ambiguity in complex organizations. *Adm. Sci. Q.* 15 150–163. 10.2307/2391486

[B61] Salin D. (2003). Ways of explaining workplace bullying: a review of enabling, motivating and precipitating structures and processes in the work environment. *Hum. Relat.* 56 1213–1232. 10.1177/00187267035610003

[B62] Samnani A.-K. Singh P. (2016). Workplace bullying: considering the interaction between individual and work environment. *J. Bus. Ethics* 139 537–549. 10.1007/s10551-015-2653-x

[B63] Schneider B. (2000). “The psychological life of organizations,” in *Handbook of Organizational Culture and Climate*, eds Ashkanasy N. M. Widerom C. P. M. Peterson M. F. (Thousand Oaks, CA: Sage)).

[B64] Schneider B. White S. S. Paul M. C. (1998). Linking service climate and customer perceptions of service quality: tests of a causal model. *J. Appl. Psychol.* 83 150–163. 10.1037//0021-9010.83.2.150 9577232

[B65] Sheehan M. McCarthy P. Barker M. Henderson M. (2001). A model for assessing the impacts and costs of workplace bullying. *Paper Presented at the Standing Conference on Organisational Symbolism (SCOS), Trinity College*, Dublin.

[B66] Siemsen E. Roth A. Oliveira P. (2010). Common method bias in regression models with linear, quadratic, and interaction effects. *Organ. Res. Methods* 13 456–476. 10.1177/1094428109351241

[B67] Skogstad A. Torsheim T. Einarsen S. Hauge L. J. (2011). Testing the work environment hypothesis of bullying on a group level of analysis: psychosocial factors as precursors of observed workplace bullying. *Appl. Psychol.* 60 475–495. 10.1111/j.1464-0597.2011.00444.x

[B68] Van den Brande W. Baillien E. De Witte H. Vander Elst T. Godderis L. (2016). The role of work stressors, coping strategies and coping resources in the process of workplace bullying: a systematic review and development of a comprehensive model. *Aggress. Violent Behav.* 29 61–71. 10.1016/j.avb.2016.06.004

[B69] Van den Broeck A. De Cuyper N. De Witte H. Vansteenkiste M. (2010). Not all job demands are equal: differentiating job hindrances and job challenges in the job demands–resources model. *Eur. J. Work Organ. Psychol.* 19 735–759. 10.1080/13594320903223839

[B70] Van Veldhoven M. Meijman T. (1994). *Questionnaire on the Experience and Assessment of Work: VBBA–English Version.* Amsterdam: The Foundation for Quality in Occupational Health Care.

[B71] Vartia M. (1996). The sources of bullying–psychological work environment and organizational climate. *Eur. J. Work Organ. Psychol.* 5 203–214. 10.1080/13594329608414855 17824934

[B72] Vartia M. (2001). Consequences of workplace bullying with respect to the well-being of its targets and the observers of bullying. *Scand. J. Work Environ. Health* 27 63–69. 10.5271/sjweh.588 11266149

[B73] Verkuil B. Atasayi S. Molendijk M. L. (2015). Workplace bullying and mental health: a meta-analysis on cross-sectional and longitudinal data. *PLoS One* 10:e0135225. 10.1371/journal.pone.0135225 26305785 PMC4549296

